# ‘Participation is integral’: understanding the levers and barriers to the implementation of community participation in primary healthcare: a qualitative study using normalisation process theory

**DOI:** 10.1186/s12913-019-4331-7

**Published:** 2019-07-23

**Authors:** Rachel McEvoy, Edel Tierney, Anne MacFarlane

**Affiliations:** 1 Graduate Entry Medical School, University of Limerick, Limerick, Ireland; 2 Graduate Entry Medical School, University of Limerick, Limerick, Ireland; 3 Professor of Primary Healthcare Research, Graduate Entry Medical School, And Health Research Institute, University of Limerick, Limerick, Ireland

**Keywords:** Community participation, Primary healthcare, Normalization process theory, Implementation theory, Health policy

## Abstract

**Background:**

Many international health policies recognise the World Health Organization’s (2008) vision that communities should be involved in shaping primary healthcare services. However, researchers continue to debate definitions, models, and operational challenges to community participation. Furthermore, there has been no use of implementation theory to study how community participation is introduced and embedded in primary healthcare in order to generate insights and transferrable lessons for making this so. Using Normalisation Process Theory (NPT) as a conceptual framework, this qualitative study was designed to explore the levers and barriers to the implementation of community participation in primary healthcare as a routine way of working.

**Methods:**

We conducted two qualitative studies based on a national Initiative designed to support community participation in primary care in Ireland. We had a combined multi-stakeholder purposeful sample (*n* = 72), utilising documentary evidence (study 1), semi-structured interviews (studies 1 and 2) and focus groups (study 2). Data generation and analysis were informed by Participatory Learning and Action (PLA) Research Methodology and NPT.

**Results:**

For many stakeholders, community participation in primary healthcare was a new way of working. Stakeholders did not always have a clear, shared understanding of the aims, objectives and benefits of this way of working and getting involved in a specific project sometimes provided this clarity. Drivers/champions, and strong working partnerships, were considered integral to its initiation and implementation. Participants emphasised the benefits of funding, organisational support, training and networking to enact relevant activities. Health-promoting activities and healthcare consultation/information events were generally successful, but community representation on interdisciplinary Primary Care Teams proved more challenging. Overall, participants were broadly positive about the impacts of community participation, but were concerned about the scope to sustain the work without the ‘protected’ space and resources that the national Initiative afforded.

**Conclusions:**

Despite the success of specific activities undertaken as part of a community process in Irish primary healthcare, the likelihood of this becoming a routine way of working in Ireland is low. Analysing the learning from this process using NPT provides theoretically informed recommendations that are transferrable to other settings and can be used to prospectively design and formatively evaluate community participation processes.

**Electronic supplementary material:**

The online version of this article (10.1186/s12913-019-4331-7) contains supplementary material, which is available to authorized users.

## Background

A number of international health policies recognise the WHO [1] vision that communities should be involved in shaping primary health care (PHC) services. PHC refers to the concept elaborated in the 1978 Declaration of Alma-Ata, which is based on the principles of equity, participation, inter-sectorial action, appropriate technology and a central role played by the health system. Statutory primary care services are the first point of contact and community participation is recommended to ensure that services are responsive to local needs [2]. Community participation is defined as:

*‘a process by which people are enabled to become actively and genuinely involved in defining the issues of concern to them, in making decisions about factors that affect their lives, in formulating and implementing policies, in planning, developing and delivering services and in taking action to achieve change’* [3] (p. 10).

This policy emphasis seeks a shift away from ‘service-led’ systems, where people are fitted into the pattern of provision that has developed historically, to ‘user-led or user-centred services’ [4]. This emphasis is clearly mirrored in the Irish system (see Additional file 1 for summary of Irish healthcare system). Primary Care: A New Direction [5], Action 19, states that: *‘Community participation in primary care will be strengthened by encouraging and facilitating the involvement of local community and voluntary groups in the planning and delivery of primary care services’* [5] (p. 39). The National Strategy for Service User Involvement 2008 [6], while using the term ‘service user involvement’, outlines a number of goals that relate to both individual and community activities. The ‘Joint Community Participation in Primary Care Initiative’ was developed as a means of ensuring community participation in PHC in socially disadvantaged communities (as per Goal 5 of the Strategy: described further in the Methods section). Most recently, ‘Slaintecare’ 2017 [7], and its accompanying HSE National Service Plan 2018 [8], shows a sustained emphasis on a shift from hospital-based to community care, interdisciplinary working and flexible person-centred services. This acknowledges research that shows that *‘the most effective actions to achieve greater health equity at a societal level are actions that create or reassert societal cohesion and mutual responsibility’* [9] (p.1011).

There have been many attempts to define community participation in the literature. It has been conceptualised as an intervention, something that managers are asked by policy makers to ‘do’, making participation a means to an end [10]. It has been conceptualised as a process focused on sustained active involvement of communities in service development [11]. Others argue that a standard definition is not possible nor useful: there is often a large gap between what health planners mean by community involvement in decision-making and control of resources and what community people mean [12]. Furthermore, the dynamic nature of community participation processes means that definitions may change for different stakeholders over time [12]. More recently, there have been advances in conceptualisation of community participation from middle–higher income countries. The concept of ‘communities of place’ is presented [10, 13], framing community participation as *‘collective actions that harness the socio cultural affiliation, customs values and beliefs through social interactions to influence and localise outcomes’* [14] (p.2). Advances in understanding about reasons for community participation are presented as a typology [10]. This typology presents a continuum with an emphasis on service and organisation effects at one end and an emphasis on people and society effects on the other [10]. Features of success in community participation, meaning sustained involvement over time, are identified by Taylor et al. [11]. Success requires motivation from a network of the ‘right’ people who have trust in each other. Success requires continued support from funders and other powerful decision makers and participants need to perceive benefits for themselves from their involvement [11].

As a policy ideal, however, implementation of community participation has proved problematic, and how it is operationalised and sustained in practice is not well understood [11, 15, 16].

These challenges resonate with the broader field of knowledge translation. Nutbeam [17] outlined the need to invest in research that improves our understanding of how effective health interventions should be implemented. Eccles et al. [18] have further argued that we need to see greater use of theoretical approaches in research focused on implementation, on the basis that this will offer (i) frameworks that are generalisable and that can be applied across different settings and individuals, (ii) an opportunity to build knowledge in an incremental manner and (iii) enhanced and more explicit analysis by virtue of using theoretical frameworks. While theory has been used to investigate implementation of community participation on interdisciplinary teams [19] there is, a lack of theoretically informed implementation research in the field of community participation in PHC.

Normalization Process Theory (NPT) [20, 21] is a conceptual framework (see Table 3 in the Methods section) designed to explain how participants understand and make sense of a practice (coherence), and how participants come to engage and support a new practice (cognitive participation). It also explains the factors that promote and inhibit the routine incorporation of complex health care interventions in practice (collective action), and how participants reflect on and evaluate it after a period of time (reflexive monitoring). The theory is relevant because it offers a conceptual framework about implementation processes. It has been developed from empirical studies about implementation and integration of innovation in healthcare settings and is, therefore, a grounded theory rather than a theory that has drawn on constructs in existing theories [22] . McEvoy et al. [23] have shown it to be useful as a heuristic device to enhance understanding of implementation journeys of a variety of interventions and innovations in healthcare settings. It has been used retrospectively in PHC settings [21] and it was used prospectively to investigate implementation of guidance and training initiatives (GTI) to improve communications between migrants and primary care providers [24], but not to analyse the implementation of community participation in PHC more broadly.

The aim of this paper is to report an NPT analysis of the levers and barriers to the implementation of community participation in PHC in Ireland.

## Methods

### Study design

Following Yin [25], this paper is based on an instrumental case study of a national Initiative for Joint Community Participation in Primary Care (hereafter JI: see Table 1 for a detailed description). The strength of a case study is that it enables researchers to gain a holistic view of a certain phenomenon or series of events [26]. The unit of analysis is *the process of implementing community participation* in order to explore levers and barriers to its normalisation as a routine way of working in practice. Drawing on Yin [25], NPT is employed as a conceptual framework to identify levers and barriers to implementation work.

**Table 1:** The Joint Community Participation in Primary Care Initiative (summarised from [30])

The Joint Community Participation in Primary Care Initiative funded and supported 19 demonstration projects in rural or urban areas of disadvantage across Ireland between September 2009 and April 2010 to work together and plan for the participation of excluded communities and groups in primary health care including their participation in local newly established PCTs and networks.	
Two of the 19 projects focused on specific target groups (Travellers^ia^ and the minority ethnic community). Each project site was managed by two principal applicants (PAs). One was a community representative, usually from a non-governmental organisation, and the other was an HSE representative. The PAs had joint ownership and management of the projects. The 19 projects were supported by two project co-ordinators who reported quarterly to the National Joint Initiative Steering Group.	
The lead community partners represented community organisations from organisational groupings such as Community Development Projects, Community and Voluntary Forums and other community organisations with a focus on health or that represented community members who have specific experiences of health inequalities (i.e. Travellers and minority ethnic groups).	
All 19 projects established Steering Groups. There were variations in the membership of the Steering Groups, with some limited to membership of the project partners, while others had a broader community and inter-agency membership. Project activities engaged a wide range of organisations and agencies charged with tackling social exclusion and local regeneration, including: • key stakeholders in the implementation of PCTs (e.g. PCT members, Transformation Development Officers/Primary Care Managers) • HSE personnel (e.g. Community Workers, Social Inclusion Officers Health Promotion Officers • non-statutory agencies (e.g. Community Development Projects Community & Voluntary Forums, Family Resource Centres.	
Some projects had a history of community participation with strong networks and relationships and enjoyed the continuing supportive involvement of significant people, while for others this was a new way of working. The most common activities that took place within the 19 projects included: • Developing and supporting a community representative infrastructure to feed into PCTs/networks • Developing joint plans between the HSE and community groups to support community participation on PCTs/networks • Training and support for PCTs on community participation.	

^ia^‘Traveller community’ means the community of people who are commonly called Travellers and who are identified (by both themselves and others) as people with a shared history, culture and traditions including, historically, a nomadic way of life on the island of Ireland. (Equal Status Acts). According to the All Ireland Traveller Health Study (2010), the key health issues for Travellers identified during the consultation process related to access to, participation in, and outcome of service provision

The boundaries of the case are a combination of time and place [27] and time and activity [28] (as we focus on the implementation work by stakeholders during the time frame of the nationally funded JI (2008–2010)).

Consistent with the case study approach [29], a combination of data methods was employed to generate the empirical data across two studies: documentary analysis, semi-structured interviews and focus groups (described in more detail further down).

The national JI was not theoretically informed but, following WHO [1], was defined as the *‘active participation of local people through processes of community development, which result in the empowerment of local communities to address health within a broader Framework of the social determinants of health’* [30] (p.iv). Based on the available national policies [5, 6], it was assumed that there would be better health outcomes from this.

The logic and reasoning for the funding was to support implementation of both the Primary Care Strategy 2001 and the community-orientated goals of the National Strategy for Service User Involvement 2008. The initial funding period was for community and health sector stakeholders in 19 sites to collaboratively design activities for their specific area during a two-year period. The intention was to extend this funding to support the work in these and other sites. There was no directive on what community participation would look like, and the central idea was for communities to generate this themselves based on their local needs and settings in partnership with members of the Primary Care Teams (PCTs). This corresponds to the notion of ‘communities of place’ [10, 13].

An independent evaluation was designed to support the process, providing a feedback loop of activities and lessons learned during the process to the participating sites *vis-à-vis* networking events, information bulletins and an online networking forum [30]. Collaborations with academics were subsequently fostered to conduct empirical analysis of the implementation of the JI. Additional file 2 provides an overview of the relationship between the Irish policy context, the JI and the empirical studies reported in this paper.

This paper draws on two complementary, theoretically informed qualitative studies (see Table 2). Both studies had ethical approval from the Irish College of General Practitioners Research Ethics Committee. While each study had some unique objectives and focused on particular groups of stakeholders, they shared a similar core theme of retrospectively exploring community participation in primary health care (PHC) in Ireland within the context of the JI. Taken together, they provide a comprehensive data set about levers and barriers to its implementation. The interview questions asked during the studies are provided in Additional file 3.

**Table 2:** Qualitative study details

Study 1	Perspective	Questions
Study 1 (2011–2014) was designed to focus on the drivers of the Joint Community Participation in Primary Care Initiative	From the perspective of senior management in HSE and policy actors in the Department of Health and with reference to key policy documents	▪ What are the ideal conditions for policy implementation of the Irish National Strategy for User Involvement?
		▪ What was the process of implementing the Strategy, with a focus on the drivers/champions of the Joint Initiative?
		▪ What recommendations can be made to maximise opportunities for policy implementation?
Study 2	Perspective	Questions
Study 2 (2012–2014) was designed to explore implementation of the Joint Initiative	From the perspective of community and HSE personnel ‘on the ground’	▪ What definitions of community participation were being used across sites?
		▪ How and why did stakeholders get involved in community participation projects?
		▪ What methods were used to enact community participation in primary care?
		▪ How do stakeholders evaluate the impact of community participation projects?

Both studies used Normalisation Process Theory (NPT) as a conceptual framework to inform the development of study-specific topic guides and to analyse levers and barriers to the implementation of the JI into routine PHC practice (Table 3). Additional file 3 presents the questions asked within study 1 interviews and study 2 interviews and focus groups.

**Table 3:** NPT constructs [31]

Construct	Explanation
1. Coherence	The work of sense-making and understanding that individuals and organisations have to go through in order to promote or inhibit the routine embedding of a practice.
2. Cognitive participation	The work that individuals and organisations have to go through in order to enrol individuals to engage with the new practice.
3. Collective action	The work that individuals and organisations have to do to enact the new practice.
4. Reflexive monitoring	The work inherent in the informal and formal appraisal of a new practice once it is in use, in order to assess its advantages and disadvantages, and which develops users’ comprehension of the effects of a practice.

### Sampling and recruitment

Following the principles of purposeful sampling [32], study 1 (*n* = 33) involved Principal Applicants (PAs) to the JI from both the HSE (*n* = 11) and community organisations (*n* = 14) from across 15 of the 19 project sites. Seven of the 18 members of the national JI steering group also participated, as did the author of the formative independent evaluation report.

In study 1, 12 of the PAs were uncontactable, had since retired, and/or were no longer in the same role or post, and hence declined to participate in the study. Similarly, a number of national steering group members had since retired or moved into new roles and felt they were not best placed to contribute to the aims and objectives of the study.

Study 2 (*n* = 39) involved community representatives,^1^ paid and unpaid (*n* = 26); HSE service providers working in PCTs (*n* = 6); HSE service planners and policy makers who oversee the development of PCTS (*n* = 4); and General Practitioners (GPs) (*n* = 3) (see Table 4). Four HSE and four community representatives also participated in study 1.

**Table 4:** An overview of the number of participants and data generation encounters

Study 1 (*n* = 32)	Status		Data Generation	Study 2 (*n* = 39)	Status		Data Generation
HSE Principal Applicants (PAs)	Paid	*n* = 11	One-to-one interview	Community Representatives	Paid	*n* = 13	Focus group
Community PAs	Paid	*n* = 14	One-to-one interview	Community Representatives	Unpaid	*n* = 13	Focus group
National steering Group, HSE	Paid	*n* = 4	One-to-one interview	Community Representative	Unknown	*n* = 1	Focus group
National steering Group, Community	Paid	*n* = 3	One-to-one interview	HSE personnel	Paid	*n* = 5	One-to-one interviews
Evaluator	Paid	*n* = 1	One-to-one interview	HSE policy personnel	Paid	*n* = 4	One-to-one interviews
				GPs	Paid (as GPS but not in capacity to support CP in PC)	*n* = 3	One-to-one interviews

Following written informed consent, three focus groups were completed and 14 interviews. There were 3, 16 and 4 participants in each group.

To ensure confidentiality, coding based on pseudonyms and/or case site numbers were used throughout the data coding process (see Table 5).

**Table 5:** Data coding process

The code (S1, HSE, Steering Group, 76) indicates the participant was from study 1 (S1), was employed by the HSE, and was a member of the National Joint Steering Group Committee. 76 was their assigned participant coding number.	
(S1, PA, Community, 4) indicates the participant was from study 1 (S1), was a PA to the JI from the community sector, and 4 was their assigned participant coding number.	
(S2/CS2, Community paid, Shell) indicates the participant was from study 2 (S2), case study site 2 (CS2), and was a paid community worker. Shell was the self-selected pseudonym.	

### Data generation and analysis

#### Study 1

Semi-structured interviews were conducted, transcribed and analysed following the principles of a framework analysis: familiarisation, identifying a thematic framework, indexing, charting, and mapping and interpretation [33]. The framework analysis was conducted using a two-stage approach: the first being an inductive framework analysis of emergent themes. Given the potential value of theory as a heuristic device to contribute to qualitative analysis processes [34] and the evidence that NPT is a useful for enhancing understanding of implementation work in health care [23, 35], emergent themes were subsequently mapped onto NPT’s four constructs. The mapping process relied on moving iteratively between the emergent themes and the NPT constructs to build knowledge about the application of NPT constructs to this specific topic and the project data. The nature of this mapping process is described in detail in MacFarlane & O’Reilly de Brun [22].

A documentary analysis involved the collation of all key documents written or produced about the JI [36] (Table 6). Based on the successful application of NPT in the analysis of interviews, and using the knowledge built up about the application of NPT constructs to this particular issue [22], these data were analysed following the principles of a deductive framework analysis based on NPTs four constructs. This deductive approach has been used successfully in other NPT studies, for example Gillespie et al. (2018) [37].

**Table 6:** Key documents analysed

Documents	Sets of Minutes	Number of Pages
National Joint Initiative Oversight Committee meetings (NJI)	1. 31st March 2009	3
	2. 23rd June 2009	4
	3. 7th Oct 2009	4
	4. 18th Jan 2010	6
	5. 31st March 2010	6
	6. 29th Sept. 2010	9
Total	6	32
Joint Initiative Networking Event meetings (JIN)	1. 4th Dec 2008	10
	2. 25th March 2009	11
	3. 22nd Sept 2009	10
	4. 20th Jan 2010	8
Total	4	39
Joint Initiative Evaluation Information Bulletins (JIE)	1. Sept 2009	2
	2. Oct 2009	2
	3. Nov 2009	2
	4. Jan 2010	2
	5. March 2010	2
Total	5	10
Formative Evaluation of the Joint Community Participation in Primary Care Initiative Executive Summary (FES)		21
Total		21
National Strategy for Service User Involvement 2008–2013 (NSSUI)		20
Total		20

#### Study 2

A combination of semi-structured interviews and focus groups were used to generate data. Community representatives choose focus groups as their preferred method of data generation as these research sessions were held to coincide with their usual scheduled meetings, which were both convenient and time-efficient. Interviews, however, were more convenient for HSE personnel, GPs, etc. as they could be scheduled at a time and location suited to the individual given the variances in their schedules of work.

In both the interviews and focus groups sessions, as shown in Table 7, Participatory Learning and Action (PLA) research techniques were used to stimulate data generation [38, 39]. These techniques have been previously used in PHC and have inherent visual and analytical properties that support generation and co-analysis of data with stakeholders [19, 40–42]. Table 7 provides a general description of each technique and an example of how it was incorporated into interviews and focus groups.

**Table 7:** Participatory Learning and Action research techniques

Flexible brainstorming	Flexible brainstorming is a technique used to generate as many ideas as possible related to the research question and recording them on Post-its on a large chart. It is suitable for those with low literacy as there are options to use pictures from magazines, draw pictures or have the research team write or spell words for participants if needed.	Flexible brainstorming was used to generate data in responses to questions about participants’ understanding of the meaning and value of community participation, what motivates them to get involved in this work, what do they do to enact community participation and how they evaluate the work.
Card sort	A card sort can be used to begin the process of thematic co-analysis of the data developed in a flexible brainstorm. All information generated during flexible brainstorming is examined and organised by asking *‘what ideas belong together? How would you organise these so that they can be organised into meaningful “bundles”?’* Participants can move the material from the flexible brainstorming chart into themes, all the while explaining why these ideas belonged together and cross-checking with each other that they are satisfied with this organisation of ideas.	The card sort was used in focus groups to co-analyse the data generated in the flexible brainstorm above.
		Responses to each question were discussed individually and organised into themes or ‘bundles’ of Post-its and pictures. Participants were asked to discuss *‘how do these ideas in question 1 fit together? What ideas belong together? what ideas are different? How can we group them together?’*
		In this way participants were co-analysing the data with each other and the researcher.

Data from interviews and focus groups were analysed according to the principles of framework analysis. As before, given the successful use of NPT in the coding of interviews in study 1, this was based on a deductive framework using NPT’s four constructs. NVivo 10 software was used to facilitate data handling and the sharing of data across the research teams.

### Quality and rigour

A number of strategies were used to enhance the quality and rigour of the analysis within each study and for the combined analysis of data. Member checking was used [43] in study 1 so all stakeholders had the chance to view their transcripts. A meeting to review findings and interpretations was held for study 2 with stakeholders in each case study area.

The researchers for each study (RM and ET) kept reflective memos/diaries recording observational notes and interactional details (e.g. of focus group discussions) to feed into the analysis process. Independent coding of transcripts was conducted to explore the development of inductive themes (study 1) and the application of deductive ones (study 1 and study 2).

As recommended with all qualitative research [44], the research team worked in a group throughout the analysis process, comparing coding, discussing thoughts about how the data related to the NPT coding frame, and refining the coding frame. Data analysis clinics were used to strengthen the initial inductive analysis process and the mapping of themes onto NPT. The authors debated the development of coding descriptors for each NPT construct and the accuracy of mapping of data onto NPT constructs, and were alert to any data that might fall outside NPT. Data analysis clinics were also used to support all deductive coding.

Taken together, this represented a reflexive approach to the analysis, which involved reflection on self, process and representation, critically examining power relations and politics in the research process and researcher accountability in data collection and interpretation including the use of theory [22, 45].

## Results

Overall, our data speak to the full range of projects activities highlighted in the formative evaluation of the JI [30], from basic health promoting interventions (e.g. drug and alcohol awareness programmes, suicide prevention programmes) to community needs assessments and representation on PCTs. These kinds of public health activities were progressed within the broader community development process and, if appropriately acknowledged and supported, could undoubtedly be used to help shape primary health care. Findings about their implementation are presented here framed around NPT’s four constructs: coherence, cognitive participation, collective action and reflexive monitoring.

### Coherence

There were shared perceptions about the main aim of community participation in PHC across all participants. Participants generally described community participation in PHC as being about developing partnerships, collaborating with all relevant inter-agency stakeholders, building trust between the community and the HSE, and ensuring that the voice of the local community was heard and involved in the design and development of local health services:

‘But with community participation it’s an equal partnership so people are coming together to see how they can maximise health outcomes in an area by working together’ (S2/CS1, HSE Personnel Paid, Digitalis).

‘Yeah to start a dialogue I suppose between community and between the statutory services and also then GPs so that the services would be delivered in a way that would be better to people who needed them the most …’ (S1, Interagency Partner Steering Group, 77).

However, at the same time it was clear that there was considerable confusion within and across stakeholder groups about what exactly was involved in this way of working. This was often a function of the range of disciplines, language and terminology used across agencies and settings. For example, according to analysis of meeting minutes and interview data, there was continuous debate about the precise meaning of the terms community participation versus service user involvement. There was also confusion when terms with different meanings were used interchangeably (NJI/31032209; JIN/04122008; JIE/092009):

‘… Some people think it’s [community participation] about lobbying, some people think it’s about developing services. Some people think it’s about developing employment initiatives. Some people think it’s about something else … and the community people are coming from one perspective, individuals come from another perspective. The primary care staff coming from a different perspective’ (S2/CS2; HSE Primary Care Development Officer, Paddy).

‘I think community, obviously it comes down to you, a lot of it as well in terms of even language, that language that is used in the community development sector, and the language that is used within the HSE, the same word can mean different things to different people and that can create sometimes some unnecessary stuff [confusion]’ (S1, PA, Community, 4).

‘She also remarked on how it struck her how we all have very different languages and the need to simplify the language and letters that we use and to demystify what it is we are referring to both within the HSE and the community’ (JIN/20012010).

Interestingly, for community stakeholders the difficulty was often in clarifying what is meant by PHC; this was not a familiar term to the ‘average’ lay person from the community.

Overall, this confusion about the meaning of community participation meant that it was hard to engage stakeholders from their wider networks in the JI:

‘Understanding what community participation involves: Yeah, on all sides I think it was very difficult to sell to people what it [community participation] involved … our challenge going forward is to continuously look at ways to see how people could have a better understanding of that …’ (S1, PA, Community, 4).

Indeed, some participants, particularly those new to enacting this way of working, openly admitted that initially they did not know the type of work that it would entail when they agreed to get involved. However, they recognised the potential opportunity to work with others who they thought had the vision, knowledge and support to drive this way of working forward:

‘I didn’t have that vision, when [HSE rep] came to me, it wasn’t my vision. I didn’t understand it the way HSE rep understood it. You know and then even applying for the Building Healthy Communities funding, like [HSE rep] very much supported us doing that because you, you know, you need a certain amount of vision at that stage with something very, very new to you, and it was very, very new to me as a community worker in the area’ (S1, PA, Community, 6).

Overall, participants were keen to highlight that it was not wise to ever assume a shared understanding of this way of working among all stakeholders.

### **‘**Cognitive participation**’**

The main drivers or champions were the PAs within the JI. They led the development of project steering groups to get the work started in each local setting (JIE/092009; FES). As illustrated in the previous quote, they were crucial in influencing stakeholders to become involved and to stay involved in the JI.

In addition, community and HSE personnel ‘on the ground’ had a key role in developing strong working partnerships between community and health service personnel:

‘Just thinking participation is integral … you need the community support and that’s the catalyst for anything to change; if you haven’t got the community backing you up and supporting things you are attempting to do you can have all the great ideas on earth but they won’t work unless they are integral to the life of the community and wanted and needed and researched valued …’ (S2/CS6; Community Representative, Laura).

The policy context was also important: HSE participants felt it was legitimate for them to be involved in the JI because this work formed part of the overall national primary healthcare strategy which was launched in 2001. The JI was viewed as an opportunity for community and HSE partners to organise themselves to collectively contribute to the work involved in implementing community participation in PHC:

‘… I wanted to see more of a role for primary care itself in the general scheme of things, because for so long we’ve talked about primary care as being the kind of cornerstone and so, and yet in practice it hasn’t always been the case …’ (S1, HSE, Steering Group, 78).

Even so, some participants explained that it was difficult to enrol other colleagues in the HSE to get involved in the work as it developed, particularly those with a clinical role, who did not always consider community participation in PHC to be part ‘of their day job’:

‘I think people were quite polite sometimes, maybe they didn’t say what they actually thought, you know, that this wasn’t, they didn’t see this as part of their work you know’ (S1, PA HSE, 14).

‘One of the biggest issues for us was staff feeling that it wasn’t their day job to engage in the Joint Initiative with the … our clinicians are saying well my job is to see people … and the facilitators have been having meetings with heads of services about you know getting heads of services agreements that this is part of their work and creating that space for clinicians that they feel ok doing it so that’s going on at the moment’ (S1, PA HSE, 6).

Another challenge cited was the issue of ‘representativeness’. HSE stakeholders in particular were often cynical about community representatives’ role. They were concerned that community representatives would only present their individual and personal views and or/issues. Most community representatives interviewed, however, did in fact understand the importance of representing the wider community rather than their own personal interests when engaging in community participation in PHC:

‘… and even though I knew it wouldn’t be just representing mental health I felt I could be a voice for them [the community] as well on the team. I suppose that was my expertise, I could give my experience from working in the mental health area and I suppose I do push that a bit. But at the same time you are still very aware that you are, it’s not just mental health issues that we discuss’ (S2/CS1, Community unpaid, Tess).

These community representatives emphasised that they believed that it was right for them to be involved in community participation in PHC, because of their training, role, responsibilities and/or personal backgrounds. Moreover, they felt they had a significant contribution to make:

‘… I grew through it, I came in as a community development worker and it was regeneration. And I live locally; I don’t live in [place name]. I live in [place name] so the whole area for me is very important how it develops. So I’m passionate from that point of view as well, it’s my area, do you know what I mean …’ (S2/CS1, Community paid, Roisin).

### Collective action

It was clear that the resources provided by the JI were pivotal to supporting the drivers and champions to enact the work. As well as having resources to establish the aforementioned steering groups, projects in the Joint Community Participation in Primary Care (JI) were provided with nationally co-ordinated networking events, community development training provided by the Community Action Network (CAN) and training in facilitation skills. The training and resources provided enabled those involved to perform the tasks required and, subsequently, provided stakeholders with a level of coherence that they may not have had prior to their involvement in a specific project:

‘There has been a huge learning curve for local community groups and primary health care staff. For the majority of community groups this has been the first time that they have formally engaged with health service providers, and the first time that primary health care staff have engaged and consulted with local communities on health issues. The projects have created a genuine sense of excitement and momentum to the ongoing work of Primary Care Teams. There have been significant benefits to joint working. The relationship building and the sharing of knowledge and information have been eye openers for many people. Primary health care staff have developed a better knowledge of the health issues facing local communities and also of the supports, networking and services that local communities can provide’ (JIE/112019).

Interestingly, with regard to training, it was striking that participants emphasised the need for training community and health professionals together. In practice, this was rare, as logistically it was often difficult to arrange times and venues convenient to both.

Participants from both the HSE and the community were very clear that macro level organisational policy and managerial support were also crucial resources for enactment:

‘and from a national point of view we’re trying to keep our heads above water trying to sustain services, trying to look at community participation, trying to do it, it’s never been sexy, it’s not one of the things that will gather their attention, at the end of the day you won’t be in front of the PAC [Public Accounts Committee] because you didn’t have community participation’ (S1, HSE Steering Group, 75).

‘I think it needs to be on the national agenda and shouldn’t be having to be reminded, that you have to remind somebody you know and to push all the time to get the Key Performance Indicator [KPI] in, like there has to be a time when it’s just part of the strategy, part of it like everything else, part of the implementation you know it’s on the same par as whatever, getting the teams [PCTs] set up getting ICT in place, getting the buildings organised, getting needs assessments done, you know, whatever’ (S1, PA HSE, 6).

At the same time, participants described meso and micro level influences on the work. Differences in working styles between stakeholders as per individual professional training meant that interactions were problematic: there were clashes between GPs’ norms around short, task-oriented meetings and community development workers’ norms around long, process-centred workshop events:

‘… they’re all coming from a different perspective, you have the private view that the fact the GPs they don’t, they really are anti any kind of long meetings or training so that’s all militating against you know …’ (S1, PA HSE, 6).

‘… not everybody liked it though, because I also remember like one of the Public Health Nurses saying she hated it … And I know that’s because, some people that, community is very process orientated, it’s very talk orientated, sometimes probably way too much! I don’t know, I suppose I feel people in the HSE services are very task orientated …’ (S1, PA Community, 13).

Overall, the most successful types of projects were those that tended to be specific, time-bound activities that resulted in a positive outcome in a relatively short period of time; for example, a health fair day, development of a directory of local community services, a one-off information/community consultation event. Some of the HSE PAs were of the view that the shorter time frame, with clear start and finish dates, was something that health professionals, particularly those with a clinical role, could and would commit to.

In contrast, longer-term activities that required ongoing support were more challenging. These included project steering groups, and community health forums^2^ (CHFs), particularly designed to support the involvement of community representatives in newly established PCT meetings.

Sites that did report successful CHFs and community representation on interdisciplinary PCTs tended to be ones where there was a strong history and experience of community participation in PHC prior to the JI. These sites had strong consistent drivers, and had invested the necessary resources over time to nourish relationships and skill sets among all stakeholders involved:

‘… I would say like with the people we’re used to working with [in the community] … that it’s very easy to work with them. But that’s taken time …’ (S1, PA HSE, 11).

‘… if I had to say what was the one most important thing that actually moved this on, that actually led to a good infrastructure in terms of the community and the HSE and particularly around primary care and the primary care teams and stuff, I would say it was that long-term working relationship’ (S1, PA Community, 6).

Finally, all participants from HSE and community sectors spoke time and time again of the importance of the aforementioned paid ‘ring-fenced’ role dedicated to initiating and sustaining the work of community participation in PHC. It was not considered sufficient that it be driven by people’s goodwill. Participants were also adamant that it was not simply just about having a named person with a ring-fenced role; it was about having a named person who is clear about the meaning of community participation and PHC, who possesses the right skill set and interpersonal qualities: ‘capacity, energy and … enthusiasm’ (S1, E, 81) are considered key.

### Reflexive monitoring

Broadly speaking, most participants reflected positively about the impacts of this way of working during the lifetime of the JI. Drawing primarily on their own informal appraisals, participants spoke of skills development as a result of training, improved networking and information sharing between community and HSE settings, and new and improved working relationships between the HSE and the community.

Participants were able to support their positive informal appraisals with several concrete examples of outcomes from their work addressing real-life local issues, including successful community health fair days, a useful information directory of community and health services, and improved signage to local health services. Interestingly, another positive outcome was that, for some, getting involved in a specific community participation project enhanced understanding of the work and its relevance:

‘Community groups have learnt about the services available, and in some cases have influenced service provision, while they have also seen the connection between the role and importance of primary health care services to the broader social determinants of health’ (JIE/112009).

In terms of more formal appraisals, the documentary analysis (FES, see Table 6), revealed that all projects initially put in place a strategy to sustain community participation in primary care in the light of the ending of the funding from the JI. Certainly there were examples from the interviews with participants of work that was sustained and further developed over time:

‘Well I think an ongoing thing that’s impressed me is that the joint meetings between the Community Health Forum and the Primary Health Care Team are ongoing … like you’re not getting enormous numbers but there is still, that channel of communication still exists’ (S1, PA Community, 11).

‘… the other thing was that it was evaluated as, coming out really well [was] the sessions [at] lunch time, hourly health-related information sessions are still there, they’re still being run, people are still coming together …’ (S1, PA HSE, 13).

However, it is important to note that a couple of participants, particularly from the community sector, were somewhat critical of the degree of progress made during the JI. One key barrier to sustainability was poor coherence – not having shared aims and objectives and expectations:

‘I don’t think we’ve managed to reach out and quite achieve everything that we thought we were going to achieve and wanted to achieve. I think that the very nature of the fact that we are a group of migrants means people come and go. And it made the group hard to gel and hard to move’ (S2/CS2, Community unpaid, Ella).

‘Ah no, I suppose there was frank discussion but I would just see that we still, at the end of the day, nothing has changed’ (S2/CS3, Community unpaid, John).

Another key barrier was the inter-linked challenges of political commitment and resources. The JI was launched during a period of rapid **economic growth**, but unfortunately the learning and formative evaluation from across the 19 sites was only being showcased as the effects of the global recession of 2008–2014 were affecting Ireland. This meant that the resources for community participation projects beyond the protected space of the JI were significantly reduced. In particular, participants remarked on the continuous ‘shifting sands’ regarding organisational priorities, and appointed roles and responsibilities, both in the HSE and in the community development sector. They also reflected on public apathy, the ad hoc development, resourcing and functioning of PCTs and the loss of personnel in protected paid roles.

Most participants reflected on their diminishing resources and highlighted the modifications and concessions made to their work to ensure that this way of working can continue. For some projects such modifications have been:

‘to pick a more specific piece of work to do … to have a standard sort of approach for every PCT … a basic step’ (S1, PA HSE, 14); ‘amalgamation of the steering group with the group of participants’ (S1, PA Community, 5); ‘for the Health Forum, I suppose, to take a bit more responsibility’ (S1, PA Community, 3); and ‘trying to be more proactive around, you know, giving information, sharing information rather than waiting for issues to arise’ (S1, PA HSE, 11).

Finally, a few participants remarked that it is too early in the process to determine the specific impacts of the JI:

‘I think as well because it was a short initiative … then they were just starting to get something from the statutory side and from the GPs to say, well actually there is benefits in this, you know, but there wasn’t long enough for those impacts really to be demonstrated, you know, people were grappling with doing it and working together and all the rest of it, but we hadn’t really gotten to the stage of impacts and outcomes’ (S1, Interagency Partner, Steering Group, 80).

## Discussion

### Summary of findings

Despite the ability of all participants to describe the idea of community participation in PHC, there was not a shared meaning in terms of the work involved within or across health service provider and community settings. This low coherence is a function of the diversity of terminology and practice, which, in turn, results from the diversity of disciplines and sectors involved. Low coherence influences enrolment work and enactment work, and attempts to appraise the impact of the work. In spite of this, the financial resources and organisational supports provided by the JI enabled individuals in healthcare and community settings in some areas, who have a clear vision for community participation and PHC, to develop relationships and drive a range of activities forward in partnership with each other. Furthermore, for some stakeholders getting involved in enactment work improved their understanding of what community participation was about and the kind of work it involved.

In the final analysis, however, and in the midst of a recession that put a strain on community and healthcare sectors, all participants were clear that this way of working is at risk, and they asked the key question: how can the work be sustained?

### Study limitations

This study is based on a funded national JI introduced in Ireland at a particular point in time. We recognise that in this study both the case and its context were changing over time [46]. The JI began during an economic boom and our fieldwork took place after a global recession that impacted considerably on Irish health care generally and the scope for community participation in particular. To address this, we have located and discussed findings in the most recent policy context.

The strength of our study is that it draws on multiple data sources and multiple stakeholder perspectives. The participation of more GPs in the fieldwork would have been beneficial. This was difficult because recruitment of GPs was possible in only one of the 19 sites, and when they were approached for this research, responses from those GPs were limited. This reflects the broader challenges in Irish PHC whereby GPs are self-employed (see Additional file 1) and participation in community processes and research are not resourced.

We further acknowledge the retrospective nature of the data and that for some stakeholders their memory of specific events was limited. However, the scope to compare and contrast data across settings and stakeholder groups was valuable in this regard.

The first author (RM) was employed by the HSE, and had a key role in co-ordinating the JI. While we could sometimes draw on RM’s ‘insider’ knowledge to enhance our understanding of the study context, we did not compromise our responsibility to understand participants’ accounts as their realities [34]. Our use of regular data analysis clinics was key in this regard.

Our qualitative case study analysis, using contemporary social theory, provided thick description [47], and is in line with international recommendations for implementation research [18]. The theoretical basis of our work enhances the generalisability of emergent themes for other healthcare jurisdictions [18, 48].

### Connections with existing literature

The JI was concerned with community participation as a process [3] and focused on a ‘community of place’ [10, 13]. The nature of the activities that developed with the available resources was equally focused on service and organisation effects and people and society effects [10]. They reflected micro and meso levels of PHC given the person-centred focus (in for example community gardens and social prescribing) and the focus on informed PCT working [51]. They included short-term and longer term goals but all were progressed within the broad remit of a community participation process.

The activities spoke more to the social determinants of health [52] and the importance of primary healthcare, which are also reflective of those in the Health Action Zone Initiatives in the UK [53]. The activities, albeit in various combinations, are also evident across projects presented in a recent review of empirical studies in the literature linking community participation and health outcomes [54].

Positive accounts of the JI resonate with features of success in community participation as described by Taylor et al. [11]: there were examples of sustained involvement of some stakeholders over time, high levels of personal commitment and motivation and the ‘right’ network of people involved in relationship building and activity development. The negative accounts relate to concerns about representativeness of community members, and tensions between short term, task-oriented activities versus long-term, process-oriented activities whose outcomes are less tangible and harder to quantify. Other negative accounts related to serious concerns about disruption to the supportive involvement of managers and resources [11] as the economic recession took effect. Indeed, this did devastate the scope for sustaining the work or extending the JI to other settings as originally intended. Therefore, while there were sustainability plans in place in specific sites, the scope for these to be realised was diminished because, as highlighted by Morgan, *‘Participation can be sustainable only as long as the relevant actors remain committed,* ***and*** *the sociopolitical and economic environments remain conducive, to the process’* [55] (p.223) [emphasis added]. In Ireland, Slaintecare [7], and the new plan for a National Health Fund, has been published [8]. This emphasises the need to ring-fence healthcare priorities such as the expansion of PHC [7]. This should allow for more stability in staffing which, in turn, would provide stability and support for interdisciplinary interactions for PHC service delivery and partnerships for community participation. It will be important, however, to develop mechanisms to resource GP involvement so that their engagement is possible in both community participation processes and associated research.

The advantage of analysing these positive and negative descriptive accounts using NPT is that it advances understanding of the implementation needed to embed and sustain community participation as a routine way of working in PHC. This NPT analysis shows that community participation in PHC is surrounded by confusion and debate among stakeholders involved about what this ‘thing’ is exactly. This low coherence is a function of the diversity of terminology and practice, which is, in turn, a function of the diversity of disciplines and contexts involved. The implication of low coherence for the implementation and normalisation of community participation is that it limits enrolment and enactment work, and limits attempts to appraise the impact of the community participation.

Another important finding from our NPT analysis of the implementation of community participation is that sense-making or coherence can be enhanced by experience and practice (collective action). This means that positive experiences of being involved in community participation projects can enhance coherence and become a lever to implementation work. The experience of having resources and support from management to work with the ‘right’ people and develop trusting relationships [11] helps stakeholders who are less familiar with community participation to ‘get it’, to see its advantages and to become more committed to the process.

The value of the NPT analysis presented here is that the advanced understanding of implementation work it provides can be used to generate knowledge about the ‘ideal’ conditions for implementation. This, in turn, allows the generation of theoretically informed recommendations to guide future projects prospectively. First, and in line with Taylor et al. [11], Gallivan et al. [49], and Tierney et al. [50], it is recommended that stakeholders should take time to discuss their shared and differential perspectives of community participation and determine who is the ‘community’ that is participating. These perspectives should be revisited as they can change over time [12].

Second, stakeholders should collectively explore and agree who are the right people to be involved in taking the work forward, bearing in mind the support needed ‘on the ground’ from community members and representatives and right through to middle and senior level management with national roles.

Third, it is important to maximise the opportunities for positive experiences so that these can ‘fuel’ the other forms of implementation work. Community participation should be contextual and is based on trust and relationships. Trust and relationships are continually built and developed in partnership [56]. This means that there must be appropriate, adequate and sustained organisational support and skills to enable the development of relationships [11].

Finally, formal systems for monitoring process and impacts are recommended. Given the diversity of perspectives about what community participation is, these should be developed in a participatory manner with all key stakeholders [50]. Once developed, these systems can inform formative evaluations to help identify problems and potential solutions during project cycles.

## Conclusion

In conclusion, despite the success of specific activities undertaken as part of a national initiative for community participation in Irish PHC, the likelihood of this becoming a routine way of working in Ireland is low. Analysing this using NPT provides theoretically informed recommendations that are transferrable to other settings. These can be used to prospectively design and formatively evaluate community participation processes in the future.

## Additional files

Additional file 1: The Irish Health System. Summary of the Irish health system. (PDF 9 kb)

Additional file 2: Relationship Overview. Provides an overview of the relationship between the Irish policy context, the nationally funded Community Participation in Primary Care initiative and the empirical studies reported in this paper. (PDF 136 kb)

Additional file 3: Topic Guides. Interview questions asked during study 1: study 2: questions asked during interviews and focus groups. (PDF 145 kb)

## Data Availability

The datasets used and/or analyzed during the current study are available from the corresponding author on reasonable request.

## Abbreviations

CHF: Community Health Forum
FES: Formative Evaluation of the Joint Community Participation in Primary Care
GPs: General Practitioners
HSE: Health Service Executive
JI: Initiative for Joint Community Participation in Primary Care
JIE: Joint Event Information Bulletins
JIN: Joint Initiative Networking Event Meetings
NPT: Normalisation Process Theory
PAC: Public Accounts Committee
PCT: Primary Care Team
PHC: Primary Health Care
PLA: Participatory Learning and Action
WHO: World Health Organisation

## Footnotes

1 Community representatives in this context were defined as: ‘individuals “representing”, representative, and/or “consultative” of one or more populations or affinity groups. They can be stakeholders, opinion leaders, organisers and advocates. They can serve as a platform and channel for information and voices of community, communicating ideas and concepts between community and health and social services and who hold people and processes accountable’ [4] (p. 4).

2 Community Health Forums have the objective to provide a representative group of people from the community who can offer a community voice on PCTs and through which PCTs can feed information about services back to the community. They form the basis to build the capacity, knowledge and expertise of community representatives to become ‘community experts’ in health. Community Health Forums have carried out various activities including the collection of information about issues affecting the health and well-being of local people, a forum for prioritising issues for PCTs to address and information sharing in the wider community about local service [30] (p.x).
