# Supplementary material for: ‘Participation is integral’: understanding the levers and barriers to the implementation of community participation in primary healthcare: a qualitative study using normalisation process theory
Source: BMC Health Serv Res. 2019 Jul 23;19:515. doi: 10.1186/s12913-019-4331-7 (PMC6651937; doi:10.1186/s12913-019-4331-7)
Supplement: Supplementary file 1 — The Irish Health System. Summary of the Irish health system. (PDF 9 kb) [file 12913_2019_4331_MOESM1_ESM.pdf]

Additional file 1

The Irish Healthcare System

The Health Service Executive (HSE) is a national publicly funded organisation, overseen by the Department of Health, which provides all health and social services in Ireland.

Ireland has the only European health system that does not offer universal coverage of primary care (Thomson et al., 2012). The HSE has a two-tier health care system whereby people with lower incomes (approximately one third of the population) are eligible for a General Medical Scheme and are entitled to free medical care. There is evidence of financial barriers to access, unmet need for care and relatively high user charges for primary healthcare when compared to other European countries (O'Reilly et al., 2007; Kringos et al., 2013).

The HSE has been working on the implementation of Primary Care Teams (PCTs) since 2001. The goal is for PCTs to comprise a wide range of health professionals, located in a single primary care centre with a wider primary care network to support the team. The HSE employs all primary health care professionals on PCTs, apart from General Practitioners (GPs) and some practice staff (e.g. practice nurses, receptionists). Most GPs are self-employed and have contracts with the HSE to provide services to those eligible for publicly funded primary care. Implementation of PCTs as a routine way of working has been slow, and many communities do not have functioning interdisciplinary PCTs in their areas.

Kringos D, Boerma W, Y Bourgueil Y et al. (2013). **The strength of primary care in Europe: an international comparative study**. *Br J Gen Pract 2013,* 63(616): e742-750.

O'Reilly D, O'Dowd T, Galway KJ et al. (2007). **Consultation charges in Ireland deter a large proportion of patients from seeing the GP: results of a cross-sectional survey**. *Eur J Gen Pract. 2007*,13(4): 231-236.

Thomson S, Jowett M, Mladovsky P et al. *Health System Responses to Financial Pressures in Ireland: Policy Options in an International Context*. Brussels, World Health Organisation and European Observatory on Health Systems and Policies; 2012.
