# Supplementary material for: ‘Participation is integral’: understanding the levers and barriers to the implementation of community participation in primary healthcare: a qualitative study using normalisation process theory
Source: BMC Health Serv Res. 2019 Jul 23;19:515. doi: 10.1186/s12913-019-4331-7 (PMC6651937; doi:10.1186/s12913-019-4331-7)
Supplement: Supplementary file 2 — Relationship Overview. Provides an overview of the relationship between the Irish policy context, the nationally funded Community Participation in Primary Care initiative and the empirical studies reported in this paper. (PDF 136 kb) [file 12913_2019_4331_MOESM2_ESM.pdf]

Additional file 2

Overview of the relationship between the Irish policy context, the Joint Community Participation in Primary Care Initiative and the empirical studies reported in the paper.

**National Primary Care Policy 2001**	**National Strategy in Service User Involvement 2008-2013**	**Joint Community Participation in Primary Care Initiative 2008-2010**	**Evaluation 2009-2010**	**HSE and Academic Research Collaboration 2010-2014**
'Community participation in primary care will be strengthened by encouraging and facilitating the involvement of local community and voluntary groups in the planning and delivery of primary health care services'	Whilst couched in the term 'service user involvement', the **seven goals** outlined in this strategy document relate to three levels of involvement: individual, community and national.<br><br>**Goal 5 of the National Strategy for Service User Involvement in the Irish Health Services:** Involvement of children, young people and socially excluded groups:	A national initiative designed to enable disadvantaged communities to participate in Primary Care (2008-2010).<br><br>Developed in partnership with Combat Poverty Agency and aimed to support and test models of community participation in primary care in 19 sites with a view to informing national policy and practice.	Independent formative evaluation of the Joint Initiative (2009-2010)<br><br>The aim of the formative evaluation was to develop and progress community participation in primary care by focusing on:<br>• The building of community infrastructure in the Initiative<br>• The project work plans, delivery and project outcomes<br>• The development of participatory methods and strategies<br>• The learning from the 19 projects across the Initiative<br>• The potential for the mainstreaming	A research collaboration was formed between the University of Limerick Graduate Entry Medical School (GEMS), The Health Service Executive (HSE) National Advocacy Unit in the Directorate of Quality and Patient Safety, community partners from national settings, and academic partners from national and international settings.<br><br>The aim of the research collaboration was to study what factors promoted or inhibited community participation activities in the sites involved in the Joint Community Participation in Primary Care Initiative.<br><br>This resulted in two studies focused on<br><br>(i) HSE management and policy documentation

			of learning for policy and service delivery.	(ii) HSE and GP personnel and community members
