# Supplementary material for: ‘Participation is integral’: understanding the levers and barriers to the implementation of community participation in primary healthcare: a qualitative study using normalisation process theory
Source: BMC Health Serv Res. 2019 Jul 23;19:515. doi: 10.1186/s12913-019-4331-7 (PMC6651937; doi:10.1186/s12913-019-4331-7)
Supplement: Supplementary file 3 — Topic Guides. Interview questions asked during study 1: study 2: questions asked during interviews and focus groups. (PDF 145 kb) [file 12913_2019_4331_MOESM3_ESM.pdf]

Additional file 3: Topic Guides developed for Study 1 and Study 2

Study 1: Topic Guide developed:

The Topic Guide was phased around the following three time frames:

1. ***The Joint Initiative (2008)***

Questions:

- How would you describe your general attitude toward CP and PC in Ireland before and after the Initiative?

- What was the **rationale** behind the Joint Initiative (JI)? *(PC strategy/service user involvement strategy?)*

- What did you hope to **achieve**? *(Did the JI exceed your expectations? If no, why not and how did that make you feel? If yes, can you give me an example?)*

- What **role** did you play in the Joint Initiative, and in particular the oversight committee?

- What helped/hindered you to play the role you just described?

- Can you talk me through who the other key players were and the roles they played? What influence/expertise did they bring to the table?

- Were there shared beliefs about the meaning and consequences of the work involved?

2. ***During the project's life time (2008-2010)***

Questions:

- In terms of the other members of the oversight group; can you tell me if these players proved influential in driving and sustaining the work? If so, please tell me how? Can you give me any particular example?

- Was there anything about this group that particularly surprised you or disappointed you?

- Outside of the oversight group, who would you say are most affected by the concept of Community Participation (CP) and Primary Care(PC)? Did you think any particular professional group facilitated or inhibited its success at a local/regional level? Can you give me an example of how they facilitated or inhibited it?

- Can you talk me through what influenced how well the JI worked?

- People have spoken of the following challenges/frustrations in implementing CP and PC (flash cards identifying (i) Shifting sands, (ii) Capacity of the PCT, (iii) Commitment and priority given to CP, (iv) Roles and responsibilities, (v) Understanding what CP involves). Would you agree/disagree with any of these? *Do any of these strike a chord with you? Or were there other challenges that you think were more pertinent particularly at the national steering group level?*

- Some of the principal applicants have spoken in depth about their relationship with the PCT and it strikes me that the capacity of the PCT comes up time and time again, what do you think?

- Before moving on, is there anything else that strikes you or that you would like to comment further on?

3. ***Sustainability of the Joint Initiative to date (May 2010 – January 2012)***

Questions:

- I realise that for some the journey has been shorter than for others, but can you describe the journey you experienced after the closing conference in May 2010? (context of the person you are speaking to) life after May 2010

- At that event there was a lot of public commitment to the work; what are your thoughts on this now? (additional prompt: From interviews carried out to date, there appear to be mixed views from participants as to such commitment…). Do you think there are people championing this work? Can you provide some concrete examples?

- In 2010, the author of the Formative Evaluation suggested that if community participation in primary care is to be sustained and rolled out across all PCTs there will be a need for the following: (Can you tell me how these have been prioritised/addressed (if at all) at a national level?)

    - A strengthened national framework, including robust guidelines and indicators so that actions are implemented and reported upon

    - Dissemination of the learning from the Initiative, including easy-to-use guidance on models of community participation

    - Ongoing resources to enable community organisations to participate effectively and articulate their interests and needs, to build the capacity, skills and awareness of community participation of both HSE and community representatives, and to bring in specialist external supports and technical assistance

    - Changes in culture and practice to ensure that community participation becomes core to the PCT work, including a stronger focus on community participation methodologies and the benefits of community participation in PCT learning and development

    - More attention to the measurement of the impacts and outcomes of community participation in primary care and a method for measuring qualitative and

quantitative impacts of community participation, from a community and a PCT perspective.

- Community participation in primary care has been introduced as a Key Result Area and a Performance Indicator in the HSE's 2010 Service Plan. How effective has this been do you think in promoting and sustaining CP and PC?

- We spoke earlier of suggestions made by the author of the independent evaluation; what other suggestions (if any) can you add to this for the future implementation of community participation in primary care?

- If you were to make one key statement in relation to your experiences of CP and PCT over the last eighteen months what would it be?

Study 2: Topic Guide developed for Focus Group and Interviews:

The Topic Guide developed for both the Focus Group and Interviews was based upon the following four key questions:

Questions:

1. What definitions/understanding of community participation Service User Involvement do you use in your work?
2. How did you become involved?
3. What do you do?/Describe the work involved in Service User Involvement?
4. What are the outcomes of this work?
